# German volume training for health promotion: Acute vasopressor, pulmonary and metabolic responses

**DOI:** 10.3389/fphys.2022.1025017

**Published:** 2022-12-19

**Authors:** Brandon S. Shaw, Rohan Lloyd, Monica Da Silva, Donne Coetzee, Jason Moran, Sally P. W. Waterworth, Musa L. Mathunjwa, Ina Shaw

**Affiliations:** ^1^ Sport, Rehabilitation and Exercise Sciences, University of Essex, Colchester, United Kingdom; ^2^ Department of Sport and Movement Studies, University of Johannesburg, Johannesburg, South Africa; ^3^ Department of Human Movement Science, University of Zululand, Kwadlangezwa, South Africa

**Keywords:** physiological response, resistance training, single exercise session, strength training, weight training

## Abstract

Resistance training (RT) is increasingly recommended for incorporation into comprehensive fitness or “exercise as medicine” programs. However, the acute effects of RT, and especially its different sub-types, and how they impact health outcomes are not fully investigated. This study evaluated German Volume Training (GVT) (“10 set × 10 rep scheme”) for its efficacy for its use in health settings. This study utilized a randomized crossover design with subjects serving as their own controls to establish baseline values. Subjects were blinded to the study hypothesis. Subjects performed a single session of GVT or no exercise, in a randomised order separated by a 1-week washout period. Outcomes were assessed before and immediately post-exercise. GVT significantly (*p* < 0.05) decreased systolic blood pressure (SBP), diastolic blood pressure (DBP) and mean arterial pressure (MAP), but increased heart rate (HR), rate pressure product (RPP) and rating of perceived exertion (RPE). No changes were found in the measured spirometry parameters. Increases were observed in carbon dioxide production (VCO_2_) and minute ventilation (V_E_), but not respiratory exchange ratio. Post hoc analysis demonstrated that post-GVT values were significantly lower for SBP (*p* = 0.017; *d* = 1.00), DBP (*p* = 0.013; *d* = 0.90), MAP (*p* = 0.024; *d* = 1.06), and VCO_2_ (*p* = 0.009; *d* = −1.32), and significantly higher for RPP (*p* = 0.001; *d* = −3.11), RPE (*p* = 0.001; *d* = −14.14), and HR (*p* = 0.001; *d* = −3.00). This study indicates that acute GVT promotes post-exercise hypotension and is of sufficient intensity to increase both objective HR and subjective RPE intensities appropriately for use in a variety of health promotion settings.

## Introduction

Resistance training (RT) is increasingly recommended by international health organizations for incorporation into comprehensive health and fitness or “exercise as medicine” programs (Shaw et al., 2021) due to its numerous and unique benefits on overall health (Shaw & Shaw, 2008; Ho et al., 2012) and correlation to all-cause mortality (Artero et al., 2011). Uniquely, RT has been shown to increase all components of muscular fitness. As with cardiorespiratory fitness, muscular fitness, and its proxies of muscular endurance and strength, appear to provide a reflection of whole-body health and function (Artero et al., 2011). In addition to reducing all-cause mortality, RT also improves dynamic stability and functional capacity, and by doing so, improves quality of life (QoL) by enabling individuals to better perform activities of daily living (ADLs) [American Sports College of Medicine (ACSM), 2004].

One of the main nonpharmacological strategies for the prevention, control, and treatment of many circulatory conditions, such as hypertension is exercise (Shaw, Brown & Shaw, 2021). A single bout of aerobic exercise can promote a sustained reduction of blood pressure (BP) for several hours in an acute effect known as postexercise hypotension (PEH) (Kenney & Seals, 1993). In fact, it has been proposed that chronic reductions in BP following chronic exercise appear to stem from the summation of acute decreases in blood pressure that occur following a single bout of exercise (Ferraria et al., 2021). However, the acute hemodynamic responses to RT are rarely investigated (McCartney, 1999). Since RT is a combination of static and dynamic contractions, the dynamic portion invokes a “volume load” on the heart *via* large increases in both stroke volume and heart rate together with reductions in peripheral vascular resistance. During RT the sustained static contractions of even a small muscle group, present a “pressure load” to the heart characterized by a moderate increase in cardiac output, minimal change in peripheral vascular resistance, and a substantial rise in mean arterial pressure (MAP) (McCartney, 1999). However, the proportions of each varying in accordance with the degree of effort required to lift the weight (McCartney, 1999) and it may be that different design variables of RT such as load, sets, repetitions and rest intervals may affect hemodynamic responses differently.

Improvements in lung function are critical to improve an individual’s ability to utilize oxygen and remove carbon dioxide, in addition to improved pulmonary function’s role in an improved immune function (Xiong et al., 2022). While long-term cardiorespiratory effects of RT are well studied and yield contradictory results, acute cardiorespiratory responses to RT modalities have only been described in a few studies (Falz et al., 2019). Previous acute studies have demonstrated that lung function appears to be only slightly affected by exercise (Liao et al., 2015; Falz et al., 2019). Should exercise training, and indeed RT, only slightly influence acute lung function, it may be the reason that such exercise does not result in significant chronic pulmonary adaptations (Falz et al., 2019). However, it appears that pulmonary adaptations due to exercise are mainly determined by training intensity and different exercise intensities might be the cause of the erratic outcomes (Falz et al., 2019).

Adaptations to aerobic and RT are highly specific. However, it appears that RT can improve cardiorespiratory endurance (VO_2max_), although not to the extent of aerobic training. RT, as with aerobic training, increases cardiac output through improvements in maximal stroke volume and maximal heart rate, and an enhanced arteriovenous oxygen difference (a-vO_2_ diff) arising from an increased capillary density and myoglobin concentration of muscle (Ozaki et al., 2013). There are also emerging data suggesting RT may affect mitochondrial dynamics (Parry, Roberts & Kavazis, 2020). However, increases in muscle mass and blood flow following RT are other possible factors that improve VO_2max_ following a period of RT (Ozaki et al., 2013). Thus, RT may provide a unique opportunity to concurrently improve both muscular fitness such as muscle hypertrophy and VO_2max_ within a single exercise modality, that is, RT. However, research is needed to determine the extent of acute involvement of mitochondrial biogenesis and the cardiorespiratory system during different types of RT (Marín-Pagán, et al., 2020; Parry, Roberts & Kavazis, 2020) to determine if such modalities are useful in health promotion settings.

The sheer range and potential blend of RT program design variables, such as *inter alia* exercise selection, intensity, and rest interval confounds the development and use of practical and easy-to-follow RT programs that will increase health outcomes (Lawrence et al., 2014). Problematically, the physiological and physical responses, adaptations and benefits are specific to those same program design variables (Arazi et al., 2011). While strength-, and muscle endurance- RT training modes are the most popular RT modalities (Baechle & Earle, 2008; Shaw et al., 2021), each year, novel or modified RT modalities are introduced into health promotion settings. One such RT modality gaining in popularity is German Volume Training (GVT), otherwise known as the “10 set × 10 rep scheme” (Backer, 2009). GVT may provide for improvements in health outcomes as it has inter-repetition RT periods of up to 3 minutes (Backer, 2009), allowing for full recovery before moving onto the next major muscle group, aiding in the ability to train with increased force for greater repetitions over multiple sets (Schoenfeld, 2010). The optimization of strength by using higher loads and fewer repetitions, as utilized in GVT, is becoming increasingly important when working with symptomatic or at-risk asymptomatic individuals with established disease, due to enhanced improvements in health and strength’s correlation to all-cause mortality (Wilborn et al., 2004; Keteyian et al., 2014). Further, RT, such as GVT, using self-selected intensity levels provides a time-efficient exercise modality which can increase adherence (Shaw & Shaw, 2021).

The use of RT with higher loads and fewer repetitions in health settings continues to be erroneously withheld from many individuals, possibly due to numerous existing position statements and/or exercise guidelines unnecessarily delaying RT and prescribing loads that are below what patients need for even the most basic ADLs without regard for the principles of specificity (Adams et al., 2006). These statements and guidelines advise caution due to historical studies demonstrating acute increases in cardiac output, stroke volume and resultant increased vasopressor responses (Okamoto et al., 2006). While long term or macro-studies are also needed that record adverse events, micro studies investigating the acute physiological responses and safety are also needed to demonstrate the can lead to immediate improvements in health, to address immediate safety concerns and to overcome findings of previous acute studies that prevail indicting the use of RT in clinical settings (Okamoto et al., 2006). In this regard, acute studies are increasingly consistent in their findings that RT provokes fewer signs and symptoms of myocardial ischemia than aerobic training, perhaps because of a lower heart rate and higher diastolic pressure combining to produce improved coronary artery filling (McCartney, 1998). We hypothesize that GVT will prove safe and efficacious for use in health promotion.

## Materials and methods

Nineteen apparently healthy males (mean age: 22 ± 1 year) with a normal body mass index (21.4 ± 5.1 kg per square meter (kg.m^−2^)) participated in a randomized crossover design with subjects serving as their own controls to establish baseline values. Subjects were blinded to the study hypothesis. Subjects performed a single session of GVT or no exercise, in a randomized order separated by a 1-week washout period. Outcomes were assessed before and immediately post-exercise. All experiments were approved by Institutional Review Board of the University of Johannesburg, and written consent was provided prior to data collection. Prior to inclusion in the study, subjects underwent a pre-screening process to assess their medical history, and any contraindications to exercise and/or testing were identified *via* the physical activity readiness questionnaire (PAR-Q) (Canadian Society for Exercise Physiology, 2002). Subjects were selected after meeting the inclusion criteria that required subjects to be male, aged between 18–25 years, sedentary for at least 6-months prior to the study, and free of any absolute or relative contraindications that could have prohibited exercise or impacted upon the exercise response. Since age and sex influence physiological responses and therefore performance during exercise exertion (Lavie et al., 2015), a male only sample was utilized to eliminate physiological and hormonal influence which would have compromised the findings of the present study.

At a separate scheduled introductory session, and with 48 h rest prior to the experiments, subjects were shown the proper technique for each exercise utilized in their individualized training programs and underwent an estimated 60% one-repetition (1-RM) for each of the exercises (machine shoulder press, machine bench press, latissimus dorsi pull–downs and machine leg press) to be used in the GVT protocol (Baechle & Earle, 2008).

**FIGURE 1:**
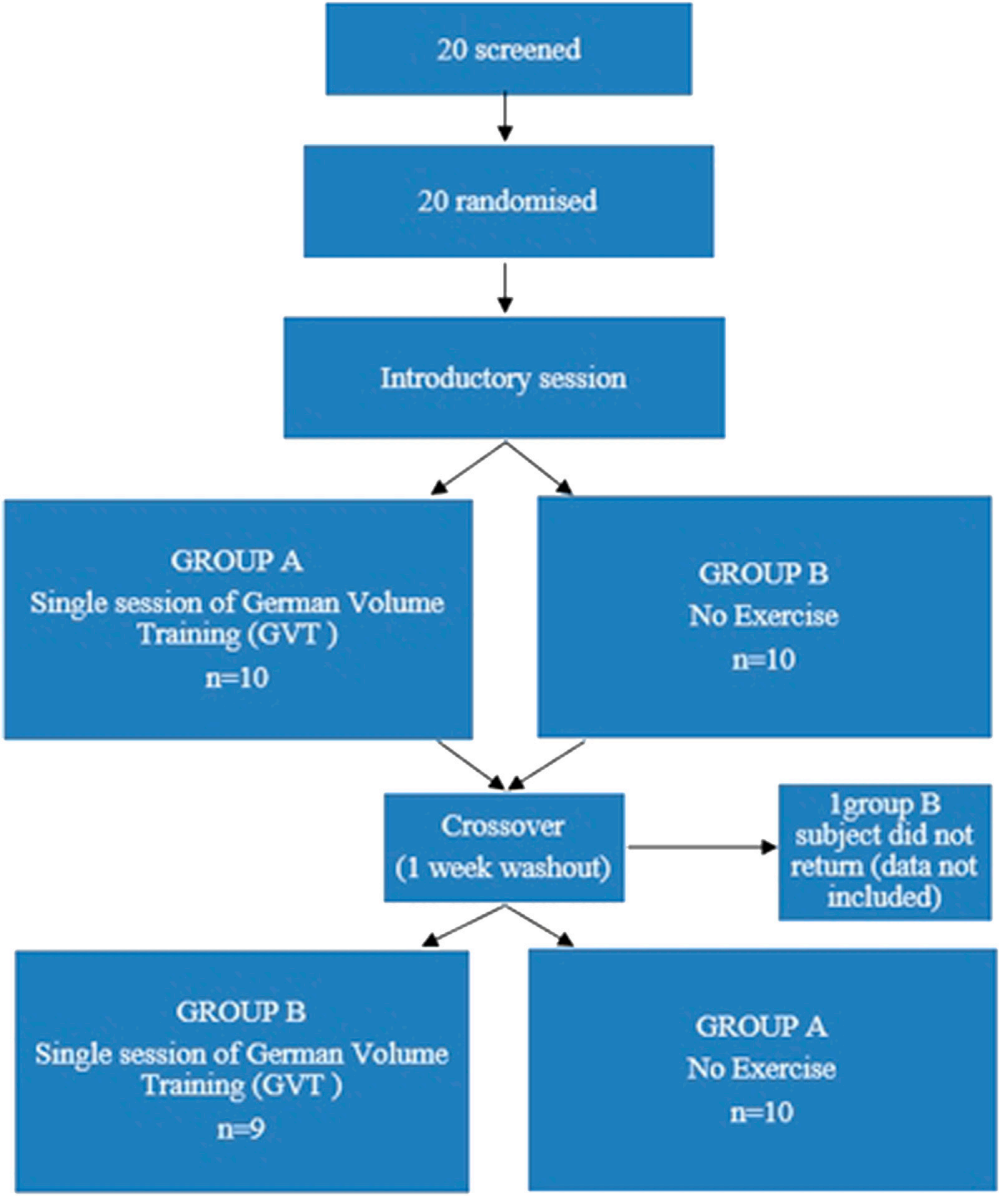
Design of randomized crossover study to examine the acute vasopressor, pulmonary and metabolic responses to German Volume Training (GVT).

After at least 48 h following the introductory session, half of the subjects were randomized in a crossover design and either performed a single session of GVT or no exercise, in a randomised order separated by a 1-week washout period. Current guidelines recommend that clinical resting measures, such as blood pressure (BP) should be optimally measured after 5–10 min of seated rest (Nikolic et al., 2014). As such, there was no need to equalize the duration of the two interventions and the non-exercise intervention required that subjects remain seated in the laboratory setting for 15 min to establish baseline data. The remaining half of the subjects then engaged in a single session of self-selected intensity GVT encompassing machine leg press, machine chest press, machine latissimus dorsi pull-downs and machine shoulder press for 10 sets of 10 repetitions each with a 60-s rest between sets and a 3-min rest period between exercises (Table 1) (Backer, 2009). Each GVT session lasted −60 min. Figure 1 provides the design of the study. Following a further 48 h, subjects then engaged in the remainder of the two experiments.

**TABLE 1:** German Volume Training (GVT) protocol.

Equipment	Volume	Rest period
Machine Leg Press	10 Sets × 10 Repetitions	60 s rest
Three-Minute Recovery Time		
Machine Bench Press	10 Sets × 10 Repetitions	60 s rest
Three-Minute Recovery Time		
Latissimus Dorsi Pull-Downs	10 Sets × 10 Repetitions	60 s rest
Three-Minute Recovery Time		
Machine Shoulder Press	10 Sets × 10 Repetitions	60 s rest

For descriptive purposes, subjects were first evaluated for body composition as per the International Society for the Advancement of Kinathropometry (ISAK) guidelines (Norton & Olds, 1996). Body mass was measured and recorded to the nearest 0.1 kg (kg) using a digital scale (PS 6600ST, Befour Inc, Saukville, WI) and body height was measured to the nearest 0.5 cm using a wall mounted stadiometer (HM210D, Charder Electronic Co, Ltd., Taichung City, Taiwan).

Heart rate (HR) and BP were measured in a seated position (Suunto t3d, Finland and Jiangsu Dengguan Medical Treatment Instrument Co. Ltd., respectively) following 5 min rest and at the completion of the baseline period (ACSM, 2018). HR was also measured in the seated position immediately following the GVT session (Cortex, MetaLyzer 3B, Leipzig, Germany). Mean arterial pressure (MAP), pulse pressure (PP) and rate pressure product (RPP) were calculated from these measures with MAP calculated using the formula: (2 x diastolic blood pressure (DBP)) + systolic blood pressure (SBP)/3. RPP was calculated using the formula: HR x SBP. PP was calculated by subtracting the DBP from the SBP (Plowman & Smith, 2011; ACSM, 2014).

Spirometry (QuarkPFTergo, Cosmed, Rome, Italy) was also measured pre- and post-experimentally for forced vital capacity (FVC), forced expiratory volume in 1 s (FEV_1_), FEV_1_/FVC ratio, mid-expiratory flow (MEF) at 25, 50%, and 75% of the FVC, for forced mid-expiratory flow (FEF25%–75%), as well as peak expiratory flow (PEF) and peak inspiratory flow (PIF) (Miller et al., 2005; Shaw & Shaw, 2011). The tests were performed at least three times with the largest value from the three tests being recorded (Shaw & Shaw, 2011).

Continuous analysis of expiratory gases was undertaken during the non-exercising control period and during the GVT protocol using an open-circuit metabolic cart (Cortex, MetaLyzer 3B, Leipzig, Germany). Oxygen consumption (VO_2_) (ℓ.min^−1^), carbon dioxide production (VCO_2_) (ℓ.min^−1^), minute ventilation (V_E_) (ℓ.min^−1^), tidal volume (V_T_) (ℓ.min^−1^), respiratory quotient (RQ) and respiratory exchange ratio (RER) were obtained at 1 minute intervals. To determine the intensity of the GVT protocol above resting levels, subjects’ direct resting metabolic rate (RMR) were also assessed while seated with little/no disturbances.

To determine the subjective intensity of the GVT treatment protocol, each subject’s rating of perceived exertion (RPE) was recorded prior to and following the non-exercising control period using the 6–20 category Borg scale (ACSM, 2014). RPE was also recorded on commencement and completion of the GVT session.

Independent samples and paired t tests were used to evaluate differences in the measured vasopressor, pulmonary and metabolic parameters between the exercise and control condition(s). Determine if any changes occurred at post-test both within- and between-groups, respectively. A two-way analysis of variance (ANOVA) for repeated measures was performed using the GVT and non-exercising treatments. Bonferroni correction was applied to correct for multiple comparisons between groups. Cohen’s *d* was further utilized to determine effect size between measures for baseline and after GVT treatment. The substantial effects for φ were divided into more fine-graded magnitudes as follows: 0.20 ≤ φ < 0.50 corresponded to a small effect size, 0.50 ≤ φ < 0.80 corresponded to a medium effect size, and φ ≥ 0.80 corresponded to a large effect size. Statistical significance was set at *p* ≤ 0.05. Data were analyzed using version 25.0 of the IBM Statistical Package for the Social Sciences (SPSS) for Windows (IBM Corporation, Armonk, NY).

## Results

In within-group analysis, a single bout of GVT significantly (*p* ≤ 0.05) decreased SBP (*p* = 0.042), DBP (*p* = 0.037), and MAP (*p* = 0.028) with a concomitant increase in RPP (*p* = 0.008) (Table 2). The single bout of GVT resulted in a significant increase in RPE (*p* = 0.007), and HR (*p* = 0.008). While no significant changes were seen in all spirometry values, increases were found in VCO_2_ (*p* = 0.021), and V_E_ (*p* = 0.021). Following the control period, significant changes were found only in MAP (*p* = 0.050).

**TABLE 2:** Acute vasopressor, pulmonary and metabolic responses to a single-session of German Volume Training (GVT).

	German volume training				Non-exercising treatment				Paired *p*-value
	Pre-test	Post-test	Indep. *p*-value^‡^	Effect size	Pre-test	Post-test	Indep. *p*-value	Effect size	
SBP (mmHg)	119 ± 9	110 ± 9	0.042∗	1.00	119 ± 9	115 ± 9	0.062	0.44	0.141
DBP (mmHg)	77 ± 10	68 ± 10	0.037∗	0.90	77 ± 10	74 ± 7	0.149	0.35	0.149
PP (mmHg)	42 ± 9	42 ± 10	0.859	0.00	42 ± 9	41 ± 8	0.670	0.12	1.000
MAP (mmHg)	91 ± 9	82 ± 8	0.028∗	1.06	91 ± 9	87 ± 7	0.050*	0.50	0.086
RPP	8370 ± 826	12332 ± 1602	0.008∗^‡^	−3.11	8370 ± 826	8281 ± 1365	0.767	0.08	0.008^†^
FVC (ℓ)	5.23 ± 0.73	5.15 ± 0.78	0.141	0.11	5.23 ± 0.73	5.24 ± 0.75	0.594	−0.01	0.260
FVC %pred (%)	104.76 ± 12.31	103.10 ± 14.10	0.173	0.13	104.76 ± 12.31	104.00 ± 12.37	0.859	0.06	0.260
FEV_1_ (ℓ)	4.40 ± 0.51	4.42 ± 0.54	0.553	−0.04	4.40 ± 0.51	4.41 ± 0.57	0.812	−0.02	0.813
FEV_1_ %pred (%)	103.88 ± 10.22	103.79 ± 10.48	0.953	0.01	103.88 ± 10.22	103.79 ± 10.48	0.953	0.01	0.812
FEV_1_/FVC	86.12 ± 8.23	87.58 ± 10.70	0.263	−0.15	86.12 ± 8.23	86.81 ± 9.1	0.086	−0.08	0.477
FEV_1_/FVC %pred (%)	103.4 ± 9.85	105.28 ± 12.83	0.260	−0.16	103.4 ± 9.85	104.36 ± 10.97	0.086	−0.09	0.441
PEF (ℓ.sec^−1^)	10.24 ± 1.85	10.93 ± 1.82	0.660	−0.38	10.24 ± 1.85	10.52 ± 1.95	0.260	−0.15	0.028^†^
PEF %pred (%)	106.8 ± 26.59	109.19 ± 18.83	0.441	−0.10	106.80 ± 26.59	105.24 ± 20.63	0.767	0.07	0.028^†^
MEF25 (ℓ.sec^−1^)	2.6 ± 0.84	2.76 ± 1.15	0.260	−0.16	2.60 ± 0.84	2.58 ± 0.97	0.553	0.02	0.086
MEF25 %pred (%)	97.64 ± 32.52	103.34 ± 43.44	0.214	−0.15	97.64 ± 32.52	96.78 ± 36.7	0.515	0.03	0.086
MEF50 (ℓ.sec^−1^)	5.45 ± 1.45	5.58 ± 1.58	0.109	−0.09	5.45 ± 1.45	5.50 ± 1.47	0.906	−0.03	0.110
MEF50 %pred (%)	98.27 ± 28.82	99.44 ± 30.14	0.374	−0.04	98.27 ± 28.82	97.98 ± 28.47	0.767	0.01	0.110
MEF75 (ℓ.sec^−1^)	8.64 ± 1.84	8.81 ± 2.09	0.374	−0.09	8.64 ± 1.84	8.58 ± 2.00	0.944	0.03	0.051
MEF75 %pred (%)	104.23 ± 26.19	103.73 ± 25.27	0.953	0.02	104.23 ± 26.19	101.11 ± 24.30	0.208	0.12	0.051
FEF25-75 (ℓ.sec^−1^)	4.85 ± 1.27	5.02 ± 1.54	0.259	−0.12	4.85 ± 1.27	4.90 ± 1.41	0.374	−0.04	0.314
FEF25-75 %pred (%)	94.02 ± 25.23	97.40 ± 30.29	0.214	−0.12	94.02 ± 25.23	95.03 ± 27.79	0.441	−0.04	0.314
PIF (ℓ.sec^−1^)	8.21 ± 3.8	8.43 ± 2.46	0.678	−0.07	8.21 ± 3.8	8.35 ± 2.31	0.859	−0.05	0.859
RPE	6 ± 0	16 ± 1	0.007∗^‡^	−14.14	6 ± 0	6 ± 0	1.000	0.00	0.007^†^
VO_2_ (ℓ.min^−1^)	0.40 ± 0.08	0.59 ± 0.24	0.066	−1.06	0.40 ± 0.08	0.37 ± 0.06	0.172	0.42	0.033^†^
VCO_2_ (ℓ.min^−1^)	0.34 ± 0.09	0.65 ± 0.32	0.021∗	−1.32	0.34 ± 0.09	0.33 ± 0.06	0.953	0.13	0.011^†^
V_E_ (ℓ.min^−1^)	17.59 ± 7.53	26.10 ± 12.60	0.021∗	−0.82	−	−	−	−	−
V_T_ (ℓ.min^−1^)	0.89 ± 0.27	1.16 ± 0.57	0.109	−0.61	−	−	−	−	−
RER/RQ	0.86 ± 0.1	0.93 ± 0.09	0.286	−0.73	0.86 ± 0.1	0.85 ± 0.07	0.859	0.12	0.173
RMR (kcal.d^−1^)	−	−	−	−	2818 ± 576	2641 ± 354	0.678	−0.37	−
HR (b.min^−1^)	70 ± 7	113 ± 19	0.008∗^‡^	-3.00	70 ± 7	72 ± 9	0.610	-0.25	0.008^†^

Values are means ± SD; *p ≤ 0.05 compared to pre-test; ^†^ *p* ≤ 0.05; ^‡^Adjusted *p* ≤ 0.01 for multiple comparisons: Bonferroni. BP, Blood pressure; mmHg, Millimetres mercury; DBP, Diastolic blood pressure; PP, Pulse pressure; MAP, Mean arterial pressure; RPP, Rate pressure product; FVC, Forced vital capacity; ℓ, Litres; FVC %pred, Percentage of predicted forced vital capacity; %, Percent; FEV_1_, Forced expiratory volume in 1 s; FEV_1_ %pred, percentage of predicted forced expiratory volume in 1 s; FEV_1_/FVC forced expiratory volume in 1 s/forced vital capacity ratio; FEV_1_/FVC %pred, Percentage of predicted forced expiratory volume in 1 s/forced vital capacity ratio; PEF, Peak expiratory flow; ℓ.sec^−1^, Liters per second; PEF %pred: Percentage of precited peak expiratory flow; MEF25, Mid-expiratory flow at 25% of the FVC; MEF25 %pred, Percentage of predicted mid-expiratory flow at 25% of the FVC; MEF50, Mid-expiratory flow at 50% of the FVC; MEF50, %pred percentage of predicted mid-expiratory flow at 50% of the FVC; MEF75, Mid-expiratory flow at 75% of the FVC; MEF75 %pred, Percentage of predicted mid-expiratory flow at 75% of the FVC; FEF25-75, Forced mid-expiratory flow; PIF, Peak inspiratory flow; RPE, Rating of perceived exertion; VO_2_, Oxygen consumption; VCO_2_, Carbon dioxide production; V_E_, Minute ventilation; V_T_, Tidal volume; RER/RQ, Respiratory exchange ratio/respiratory quotient; RMR, Resting metabolic rate; HR, Heart rate.

Significant differences were found between the two treatment conditions for RPP (*p* = 0.008), RPE (*p* = 0.007), HR (*p* = 0.008), PEF (*p* = 0.028), VCO_2_ (*p* = 0.011), and VO_2_ (*p* = 0.033). Post hoc analysis demonstrated that post-GVT values were significantly lower for SBP (*p* = 0.017; *d* = 1.00), DBP (*p* = 0.013; *d* = 0.90), MAP (*p* = 0.024; *d* = 1.06), and VCO_2_ (*p* = 0.009; *d* = −1.32), and significantly higher for RPP (*p* = 0.001; *d* = −3.11), RPE (*p* = 0.001; *d* = −14.14), and HR (*p* = 0.001; *d* = −3.00).

## Discussion

An accumulating body of work demonstrates that RT, and much of its subtypes, is both safe and efficacious. Further, while previous research has indicated that individuals differ greatly in the levels of intensity they self-select, with some choosing intensities that are too low to be effective or too high to be safe, this study demonstrates that self-selected intensity GVT results in PEH and is of sufficient intensity to prove useful in health settings.

### Hemodynamic measures

Blood pressure is routinely measured during exercise testing, health assessment, and clinical evaluation not as a measure of exercise intensity, but rather to examine exercise-induced vascular, hormonal, and neural control mechanisms crucial to the safety of individuals and necessary for assessment and interpretation of the exercise response (Griffin et al., 1997). Specifically, this study found that a single session of GVT reduced SBP, DBP, and MAP with concomitant clinically large effect sizes. This is similar to the findings of Mohebbi et al. (2009) who found that RT performed at an intensity of 40 or 80% of 1-RM, three or six sets, respectively, provoked hypotension during the recovery period. Importantly, Khant and Shah (2013) found that concentric RT, rather than eccentric RT, results in larger reductions in SBP, DBP, MAP, RPP, and HR. BP reductions or PEH following acute exercise is immediate and expected persisting for up to 24 h after an exercise bout, even in normotensives (Carpio-Rivera et al., 2016; MacDonald & Pescatello, 2018) as utilized in this study. In this study, HR and RPP were both found to be increased following the GVT session along with concomitant clinically significant large effect sizes. While this increased RPP indicates that the cardiac muscle was adequately stressed by the GVT, the RPP of 12332 ± 1602 found at post-test in this study can be considered a relatively low hemodynamic response (i.e., 10000–15000) to a total exercise session. However, to confirm a low hemodynamic response by RPP, future studies should monitor RPP at various timepoints throughout the exercise session. While previous research has found that RT increases PP (Bertovic et al., 1999), PP was unchanged in the current study, possibly due to the decreases in both SBP and DBP. Similarly, the decrease in MAP across the baseline could be explained by the sensitivity of indexes when compared to single based values, such as SBP and DBP (Shaw et al., 2015). As such, these findings support the Eighth Report of the Joint National Committee on Prevention, Detection, Evaluation, and Treatment of High Blood Pressure and the American College of Sports Medicine which recommends acute exercise as an emerging modality, as initial therapy to prevent, treat, and control hypertension in the future (MacDonald & Pescatello, 2018).

### Spirometry

Research has demonstrated that acute prolonged endurance running acutely reduces spirometric lung values (Zavorsky et al., 2019). This decrease in spiromertic values is dangerous and is associated with an increased morbidity. This is because acute deceases in spiromertic values/lung function arise from several mechanisms including exercise-induced bronchoconstriction (EIB) and/or exercise-induced respiratory muscle fatigue (Zavorsky et al., 2019). Despite the importance of monitoring lung function in acute (and long-term) exercise to scrutinize the effectiveness of exercise interventions and/or the progress of existing cardiopulmonary disease, this study is novel in its investigation of the acute effects of short-term exercise on lung-function. While previous long-term intervention studies demonstrating improvements in spirometry following anaerobic (Coppoolse et al., 1999) or RT (Shaw et al., 2011), no such changes were found in this acute study. Further acute and long-term intervention RT studies are required, in that such training more closely resembles the ADLs of individuals and patients more than aerobic training (Coppoolse et al., 1999).

### Continuous analysis of expiratory gases

In this study, a single session of GVT failed to elicit a change in VO_2_ despite a large clinically significant effect size being found. GVT increased VCO_2_ and resulted in a concomitant increase in V_E_, both of which also observed clinically significant large effects. These increases in VCO_2_ and V_E_ are expected with the onset of exercise because of the central neural respiratory generator, the sensory input system, and the muscular effector system stimulating the respiratory control system. The lack of increase in VO_2_, may be due to the GVT used in this study being considered a low hemodynamic response as confirmed by RPP. While this study is unique and no studies exist to corroborate or refute the findings, previous research on long-term RT have demonstrated increases in VO_2submax_ and VO_2max_, with simultaneous increases in V_E_ (Hackett et al., 2012). Further analysis of the mean RER of 0.93 ± 0.09 post-GVT indicates a preference for carbohydrates to be used as a fuel source during the GVT (Plowman & Smith, 2011). This opposes traditional views that would see this type of training as moderate intensity (50%–70% HR_max_), moderate duration (40–150 min) and thus should have fats, or rather, free fatty acids (FFA) as the dominating fuel source, with only moderate reliance on carbohydrates (glucose and glycogen) (Plowman & Smith, 2011). A possible explanation for this may be that while the entire GVT session used in this study lasted about 60 min, subtracting for the 60-s rests between each set, and 3 min recovery between exercise, net exercise time was only approximately 15 min. This makes GVT especially useful in that this type of training allows greater workout volume by permitting for greater consistency in repetitions over all sets (Miranda et al., 2009). This may make GVT especially useful in health promotion settings where volume of work is more important than intensity or load (Shaw et al., 2009; Shaw & Shaw, 2021).

### Subjective intensity

While previous research has noted that traditional RT recovery periods do not allow for HR and SBP to return to near- or baseline values during both low- and high-intensity RT modalities (Willardson & Burkett, 2005), GVT utilizes rest intervals of up to 3 min, which allows a higher the total number of sets (and volume) to be completed (Willardson & Burkett, 2005). Despite these relatively long rest periods, subjects perceived the entire GVT session at a mean RPE of 16 ± 1, effectively demonstrating their perception of the GVT session as “somewhat hard”. The change in RPE following GVT also yielded a clinically significant large effect size, which corresponded to the large effect in HR. This finding supports the use of RPE to quantify RT in that it may quantify the amount of total work during a RT session (Pritchett et al., 2009). Since general exercise prescriptions for health promotion propose an aerobic intensity of 11–13/20 RPE, future research should monitor RPE at various timepoints (i.e., following sets and/or exercises) throughout the exercise session and determine if RPE returns to baseline during the recovery periods or if a cumulative effect exists during the entire session.

### Limitations

There were limitations to this research, including data on outcomes are reported before and after the entire 60-min GVT session but not for the timepoints immediately after the individual exercise sets during GVT. Future studies should examine the effect of different exercises within GVT program design to determine the safety and efficacy of individual exercises to show how much the cardiovascular system is stressed during this type of training and to optimise GVT program design in a health setting. This study utilized self-selected intensity and while results indicated that the experimental GVT program resulted in PEH and is of sufficient intensity to prove useful in health settings, previous research has highlighted that individuals may differ greatly in the levels of intensity they self-select and may choose intensities that are too low to be effective or too high to be safe. As such, future studies should either directly measure or estimate 1-RM and/or HR_max_ and set intensity levels accordingly. Additionally, GVT was only compared to a resting period and not compared to a second exercise group implementing conventional RT.

In conclusion, while RT is increasingly being recommended by international health organizations for health promotion, the sheer diversity and potential combination of RT program design variables complicates the development and use of practical and easy-to-follow programs that will increase health outcomes. More specifically, the various RT modalities may elicit unique physiological and physical responses. Collectively, at a macro level, results from long-term studies, reviews and meta-analyses have suggested that RT interventions in general are safe and have the potential to favourably impact on health. By contrast, little research has focused on the benefits and safety associated with RT, especially novel or non-traditional forms, from a micro-level perspective. This necessitates that new and lesser-known RT modalities, such as GVT be investigated for its safety and efficacy in health promotion settings. Although benefits following a single bout of RT are considered transient, such findings indicate an immediate and/or faster improvement in health. In addition, new and emerging research is needed on the effects of acute exercise that has the potential to alter the way in which exercise is prescribed to prevent, treat, and control diseases in the future.

In summary, while RT design considerations can be fine-tuned by advanced exercise scientists, practical and easy-to-follow, evidence-based programs for use in general health settings are limited. Problematically, RT sub-types, and especially those with simple design principles (i.e., 10 sets by 10 reps) have not been assessed for use in health promotion settings. This study indicates that acute GVT promotes PEH and is of sufficient intensity to increase both objective HR and subjective RPE intensities appropriately for use in a variety of health promotion settings as promoted by the American College of Sports Medicine (2009).

## Data Availability

The raw data supporting the conclusions of this article will be made available by the authors, without undue reservation.
